# Black American and Latinx Parent/Caregiver Participation in Digital Health Obesity Interventions for Children: A Systematic Review

**DOI:** 10.3389/fdgth.2021.687648

**Published:** 2021-06-15

**Authors:** Jennifer Sanchez-Flack, Joanna Buscemi, Alexander O'Donnell, Margaret H. Clark Withington, Marian Fitzgibbon

**Affiliations:** ^1^Department of Pediatrics, University of Illinois at Chicago, Chicago, IL, United States; ^2^University of Illinois Cancer Center, University of Illinois at Chicago, Chicago, IL, United States; ^3^Institute for Health Research and Policy, University of Illinois at Chicago, Chicago, IL, United States; ^4^Department of Psychology, DePaul University, Chicago, IL, United States

**Keywords:** digital health, obesity, health inequities, behavioral interventions, systematic review

## Abstract

Parents/caregivers are consistently described as integral targets given their influential role in supporting and managing behaviors such as diet and physical activity. Identifying effective obesity prevention interventions to enhance and sustain parent participation is needed. Digital obesity prevention interventions are a promising strategy to improve parent/caregiver participation. Digital health interventions demonstrate acceptable participation and retention among parents/caregivers. However, our understanding of digital obesity prevention interventions targeting Black American and Latinx parents/caregivers is limited. This systematic review aims to identify Black American and Latinx parents'/caregivers' level of participation in digital obesity prevention and treatment interventions and determine the relationship between parent/caregiver participation and behavioral and weight status outcomes. This review adheres to PRISMA guidelines and is registered in PROSPERO. Eligibility criteria include: intervention delivered by digital technology, targeted Black American and Latinx parents/caregivers of young children (2–12 years), reported parent/caregiver participation outcomes, targeted diet or physical activity behaviors, and randomized controlled trial study design. Searches were conducted in September 2020 in ERIC, PsychInfo, PubMed, and Web of Science. Initial searches returned 499 results. Four reviewers screened records against eligibility criteria and 12 studies met inclusion criteria. Across all studies, parent/caregiver participation ranged from low to high. Only half of the included studies reported significant improvements in behavioral or weight status outcomes for parents/caregivers and/or children. Of these studies, three reported high parental/caregiver participation rates, and three reported high satisfaction rates. These findings suggest that participation and satisfaction may impact behavior change and weight status. The small number of studies indicates that additional research is needed to determine whether engagement or other factors predict responsiveness to the digital health intervention. Our results lay the groundwork for developing and testing future digital health interventions with the explicit goal of parental/caregiver participation and considers the need to expand our digital health intervention research methodologies to address obesity inequities among diverse families better.

## Introduction

Younger generations have earlier and longer exposure to excess adiposity over their lifetime compared to previous generations (1). This longer-term exposure to excess adiposity is problematic. Additionally, obesity is associated with hypertension (2), type 2 diabetes (3), coronary heart disease (CHD) (4), stroke (5), and osteoarthritis (6) among other chronic conditions. Recent evidence demonstrates that obesity and an obesogenic diet accelerates the transition of tissue from normal to invasive malignancy and metastatic disease (7, 8). Recent data demonstrates that obesity prevalence for youth (aged 2–19 years old) is 18.5%; with obesity prevalence among preschool-aged children (2–5 years) at 13.9 and 18.4% among school-aged children (6–11 years) (9). Additionally, obesity inequities exist, and racially/ethnically diverse children have higher rates of obesity than non-Latinx Whites. For example, 19.5% of Black American and 21.9% of Latinx children have obesity, compared to 14.7% of non-Latinx White children (10).

Strategies to prevent and treat childhood obesity include the promotion of healthy dietary and physical activity behaviors. The promotion of such behaviors is important because of the low dietary quality and increased physical inactivity of children in the United States, which is contributing to the overweight and obesity risk, the increased risk younger generations are facing for six of the 12 obesity-related cancers, and increased risk of chronic conditions such as CHD in adulthood (11, 12). The dietary quality of children has worsened in recent decades with data showing a low consumption of fruit and vegetables, whole grains, and fish and a high consumption of sodium and sugar-sweetened beverages among children (13). Similarly, children in the United States do not meet recommended guidelines for physical activity, with only half engaging in the recommended 60-min of physical activity per day (14). Currently, underserved children, such as Black American and Latinx children, demonstrate poorer dietary and physical activity patterns, as compared to non-Latinx White children, which may explain their disproportionate rates of obesity. Young children from minority and low-income communities do not meet USDA recommended dietary guidelines (15–18). For example, the California Health Interview Survey study found that Black American and Latinx children consumed more sugar-sweetened beverages, fruit juice and fast food consumption and consumed less fruits and vegetables compared to non-Latinx White children (19). Underserved children also do not meet recommended guidelines for physical activity, with only half engaging in the recommended 60-min of physical activity per day (14) and low-income Latinx children exhibiting the lowest rates of physical activity (20). Therefore, preventing or treating overweight/obesity earlier, by promoting healthier behaviors, can help reduce the lifetime risk of overweight, obesity, and obesity-related chronic diseases (21).

Children are primarily socialized within the family environment, with parents/caregivers serving as gatekeepers to lifestyle behaviors (22). Given the family's highly influential role in supporting and managing lifestyle behaviors, parents/caregivers are integral targets in health behavior interventions (23–25). Recent systematic reviews report that obesity prevention interventions for young children result in more positive changes in both weight status and obesity-related behaviors when they include a parent/caregiver participation component, compared to interventions that do not (26, 27). However, in previous obesity prevention interventions with a parent/caregiver participation component, participation has been low, but nonetheless positively associated with successful changes in children's behavior (28–30). Commonly cited barriers to participation include time constraints, lack of childcare, and lack of transportation (28–31). Therefore, effective, and efficacious obesity prevention interventions including parents/caregivers are urgently needed.

Digital health interventions (DHI) are a promising strategy to improve and maintain Black American and Latinx parent participation in obesity prevention interventions. Approximately 96% of Americans, including racially/ethnically diverse and low-income populations, own a smartphone or a cellphone, with smartphone ownership being more common (32). Additionally, Black American and Latinx populations rely more heavily on smartphones for online access or are “smartphone only” internet users, meaning they lack traditional home broadband service but do own a smartphone, compared to non-Latinx White populations (32, 33). Therefore, traditional in-person evidence-based interventions could be adapted to be digital delivery (e.g., text messages, websites, or mobile applications) to enhance participation in obesity prevention or treatment programs (34). Recent internet- and mobile-based interventions show acceptable participation (used DHI at least one time/week) (35) and retention (above 80% retention rate at post-intervention) among parents/caregivers with young children (36, 37). Although the number of DHIs among parents/caregivers of Black American and Latinx young children is limited, there is a need to systematically evaluate this body of literature to (1) determine the effectiveness of DHIs and (2) the relationship between parent/caregiver participation and behavioral and health outcomes for both parents/caregivers and children. The primary aim is to identify Black American and/or Latinx parents'/caregivers' level of participation in DHIs for obesity prevention or treatment for their children. The secondary aim is to determine the relationship between parent/caregiver participation and behavioral (diet and physical activity) and health outcomes for both parents/caregivers and children.

## Methods

### Study Design

The systematic reviews adheres to the Preferred Reporting Items for Systematic Reviews and Meta-Analyses (PRISMA) (38) and is registered in PROSPERO (ID: CRD42020194390).

### Inclusion and Exclusion Criteria

The search protocol was developed using the Population, Intervention, Comparison, Outcomes, and Study Design (PICOS) framework for systematic reviews (38). A study was included if: it was peer-reviewed, a randomized-controlled trial, a DHI targeting obesity prevention and/or treatment, participants were primarily Black American and/or Latinx (at least 25% of the study sample), included young children aged 2–12 (39) and their parents/caregivers, included measured outcomes of parent participation (e.g., user-reported interaction with the DHI through self-report questionnaires, interviews) (40, 41), and measured outcomes of dietary intake, physical activity, and/or weight status. For the purposes of this review, to meet the definition of a DHI, interventions had to use digital technology to promote and/or maintain health, including web-based strategies, mobile health applications, text messaging, automated healthcare and communication systems, or a combination of these digital technology strategies (42). Studies were excluded if they were not published in English and were not peer-reviewed.

### Search Strategy

Searches were conducted in ERIC, PsycINFO, PubMed, and Web of Science in September 2020. The search strategy was developed in consultation with and reviewed by an experienced university librarian. All search histories were documented in an Excel spreadsheet, which contained data regarding the database searched, filters, number of records retrieved and number of duplicates. Search strings corresponded to the following six terms: (1) obesity; (2) diet; (3) physical activity; (4) digital health; (5) parents; and (6) race/ethnicity and were limited to randomized controlled trials. Searches were not limited by publication date. The full search strategy for all databases is presented in Supplementary Material 1.

### Selection and Screening

**Figure 1:**
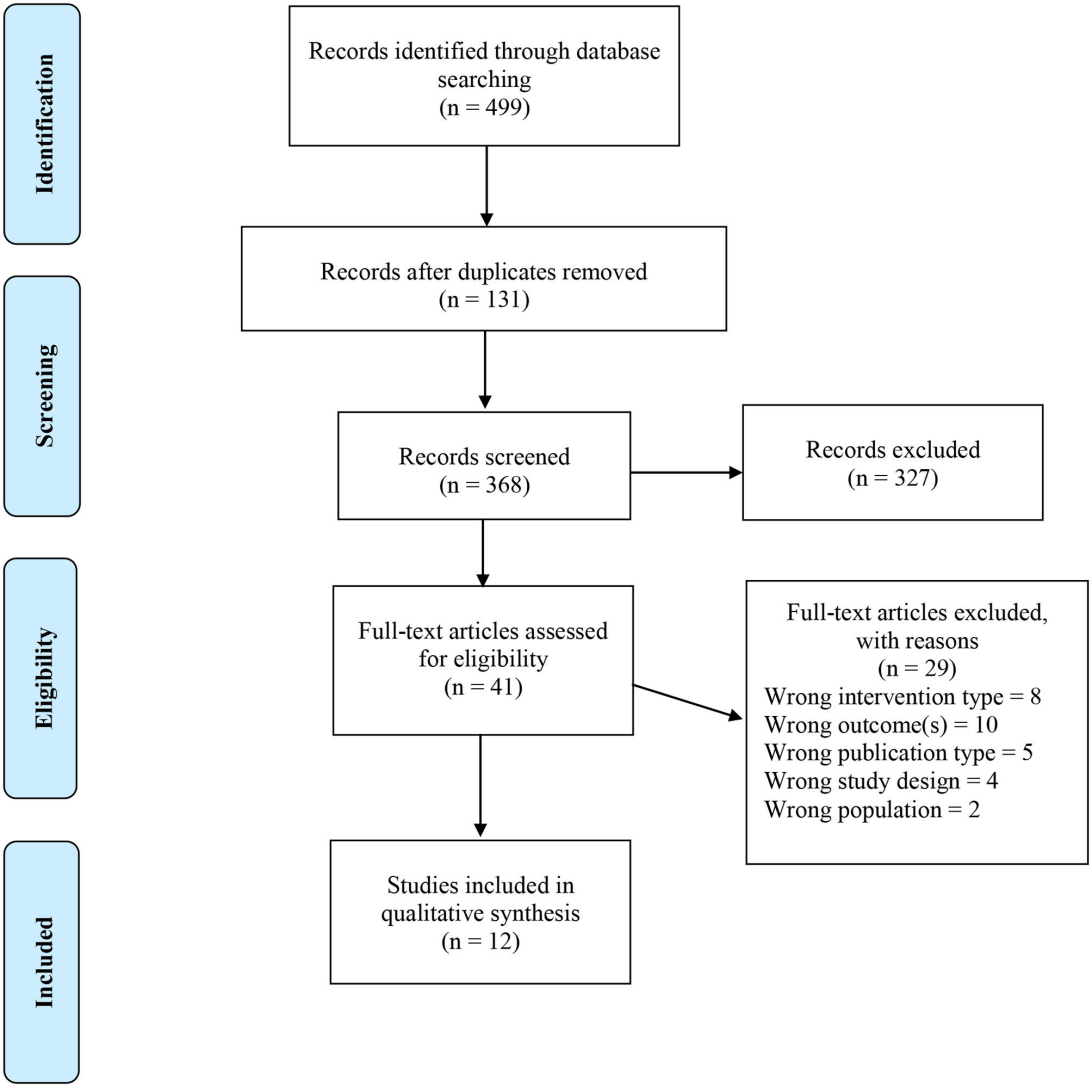
Study selection flow diagram.

All citations were imported into RefWorks for identification of duplicates. In RefWorks, separate folders were created for each database searched. First, internal duplicates (duplicates within the same database) were identified. Second, external duplicates (duplicates within separate databases) were identified and reported. Duplicates not identified within RefWorks, were identified in Covidence–a program developed for managing systematic review title/abstract and full text screening and data extraction (Covidence systematic review software, Veritas Health Innovation, Melbourne, Australia. Available at www.covidence.org). Figure 1 illustrates the search strategy and screening process in more detail. Initial searches resulted in 499 records and after the removal of duplicates, 368 records were included for screening in Covidence. Three reviewers (JSF, AO, and MW) independently screened records against eligibility screening in two phases: (1) title and abstract and (2) full text. In both screening phases, JSF screened all records and AO and MW screened 50% of records. Any discrepancies were resolved by an additional reviewer (JB). If an abstract was missing for any citation, the article continued onto full text screening. During title and abstract screening, 327 records were excluded, which resulted in 41 full text articles to be assessed for inclusion. Upon completion of full text of screening, 29 were excluded resulting in 12 studies to be included.

### Data Extraction and Synthesis

Data were extracted into a form developed by JSF. The data extraction form was determined at the outset of the study, based on study aims, and the form was piloted on a small sample of studies. Then, JSF extracted 100% of the data, AO extracted data from 50% of studies and MW extracted data from the other 50% of studies. When data extraction was completed, JB compared all extracted data and resolved all discrepancies, which were identified in Covidence as highlighted data extraction discrepancies between JSF, AO, and MW. The following information was extracted: author, year of publication, city/state/country, setting, sample size, participant characteristics (e.g., age, race/ethnicity, inclusion, and exclusion criteria), type of DHI (e.g., web-based, text message), brief description of DHI, DHI duration, diet and/or physical activity behavioral outcome measures, parent/caregiver participation measures, parent/caregiver participation outcomes, retention rate/withdrawals/loss to follow-up, diet and/or physical activity outcomes for parent/caregiver and/or child. The categories for data extraction were kept broad because of methodological differences across studies (43). A narrative synthesis, specifically a textual narrative synthesis, of studies meeting the inclusion criteria was conducted (44, 45). A narrative synthesis is a systematic review approach that relies primarily on the use of text to synthesize findings from multiple studies to summarize and explain findings; it is best used when statistical meta-analysis is not feasible due to considerable methodological and clinical differences between studies (46). Study characteristics, DHI strategies, measures, and findings are reported according to a standard format and similarities and differences are compared across studies (47). Bias ratings were conducted in accordance with the guidelines of the Cochrane risk of bias assessment tool (48), which outlines qualifications for high, low, or unclear risk of bias.

## Results

### Description of Studies, DHI Strategies, and Dose

Study design characteristics, including a brief description of the DHI, sample size and description, study duration and DHI dose, and outcome measures are reported in Table 1. Of the 12 studies, all were conducted in the United States, the sample sizes for the studies ranged from 27 to 721 participants or parent/child dyads, the age range of children included in the studies was between 2 and 15 years old, and study duration ranged from 3 weeks to 2 years. All the included studies included Black American families in their sample and three studies included Latinx families. Of these studies, 28–100% of the study sample identified as Black American and 7–51% of the study sample identified as Latinx. The current review included both obesity prevention and treatment DHIs. Types of DHI strategies included text messages, websites, e-mails and Interactive Voice Technology (IVR). Of the included studies, four were obesity treatment interventions and recruited children with overweight and/or obesity (49, 57, 59, 60), one study was an obesity prevention intervention that recruited mothers with overweight/obesity (55), and the remaining studies were obesity prevention interventions that recruited children and parents/caregivers regardless of weight status (50–54, 56, 58). Nine studies were rated as low risk for bias (49–51, 53, 55–58, 60), two studies were rated as unclear (52, 59), and one study was rated as high risk (54).

**Table 1:** Characteristics of studies included in systematic review.

**Study (Author name, year, and city/state, risk of bias)**	**Sample size**	**Characteristics of population**	**DHI methodology**	**Brief description of DHI**	**Duration and Dose**	**Behavioral and weight status measures**
Armstrong et al., 2018, Durham, NC, USA, low risk of bias (49)	*N* = 100	Children aged 5–12 years old and their parent/guardian enrolling in tertiary-care obesity treatment. 48% of participants identified as Black American.	Text messages.	Usual care plus daily text messages to parents. Text messages consisted of 100 Motivational Interviewing (MI) prompts. The week's first text message persuaded parents/guardians to identify and set a family health goal. Reply text messages reinforced evidence-based goals that were likely to lead to the reduction of child BMI. Each week, parents/guardians were invited to choose a new goal or continue working on the previously selected goal. Text messages were sent on weekdays at 12 pm and replies to parents' responses were twice/day and sent by 5 pm. Appointment reminders were sent by text 24–48 h before a scheduled usual care appointment.	Duration: 12-weeks Dose: First week included three text messages and three parent replies. Subsequent weeks included 1–2 text messages and 1–2 parent responses.	- Parent/Guardian: Body Mass Index (BMI). - Child: Food Frequency Questionnaire (FFQ), BMI, physical activity, screen time.
Baranowski et al., 2003, Houston, TX, USA, low risk of bias (50)	*N =* 35	Black American 8-year-old girls attending summer camp and one of their parents/guardians.	Website with email reminders.	Website consisted of weekly behavioral or environmental goals for children and parents/guardians. Children's webiste included: (1) comic book with summer camp characters who overcame barriers in making lifestyle changes consistent with diet and physical activity goals; (2) problem solving for diet and physical activity barriers; (3) review of previous week's goals; (4) opportunities to set new diet and/or physical activity goals; (5) photo album from the camp; (6) ask the expert feature; and (7) links to various websites. If children did not click on a webpage item within 10-s, items on the page began to flash, to encourage a click. Parent/Guardian website included: (1) comic book where a character commented on each frame of the child's comic; (2) a poll regarding the best methods to support lifestyle changes in their children with feedback from all parents the following week; (3) opportunity to set a goal to help their children make a lifestyle change each week; (4) review of previous week's goal attainment for parents and children; (5) ask the expert feature; (6) links to various websites; (7) link to their child's website.	Duration: Summer, July-August - Dose: Website updated and e-mail reminders sent weekly.	- Child: 24-h dietary recall, accelerometer, physical activity questionnaire, waist circumference, body fat percentage (DEXA scan), BMI.
Cullen et al., 2017, Houston, TX, USA, low risk of bias (51)	*N =* 126	Black American families with 8–12-year-old children, with access to a home computer with high-speed Internet.	Website with video stories.	The 8 stories follow an Black American family with two 8–12-year-old children as they try to develop healthier dietary habits. After viewing the video story, parents/guardians set a goal for the next week and viewed a family food problem. Parents/Guardians provided their opinion on how to solve the food problem via a website poll. The following week, parents/guardians viewed poll results and recorded whether they met their goal. Session content and recipes could be downloaded from the website. Session content included: (1) deciding behavior change; (2) getting started; (3) menu planning at home; (4) eating away from home; (5) recipe modification; (6) grocery shopping; (7) healthy food prep practices; (8) maintaining healthy family eating habits.	Duration: 2-months. - Dose: Weekly; parents/guardians could only view one session/week but could view other materials and watch the video story as needed.	- Parent/Guardian: Dietary behaviors, home availability of fruit, vegetables, and high-, low-, and fat-free foods. - Child: Fruit and vegetable intake.
Frenn et al., 2013, Midwest, USA, unclear risk of bias (52)	*N =* 62 dyads	Low- to middle-income 5th, 7th, and 8th grade students and one parent/guardian from three urban schools. 28% of students identified as Black American and 7% identified as Latinx.	Website.	Parent/Guardian intervention: 6-modules to teach parents/guardians effective authoritative parenting, strategies to provide positive reinforcement for healthy eating and physical activity, and role modeling healthy behaviors. Opportunities to participate in online discussions, websites for family outings, and recipes provided on website. Child intervention: Four 2–3-min videos with diverse child actors from similar schools. Interactive components, additional websites, and ideas on recipes children could make with parent/guardian.	Duration: 3–4 weeks. - Dose: Self-paced; each parent/guardian module took 5–10 min to complete; each child module took 10–30 min to complete.	- Parent/Guardian: BMI, family support for reduction in dietary fat, sedentary behavior, and physical activity, and Food/Activity Parenting Practices Questionnaire. - Child: BMI, dietary fat, physical activity.
Haines et al., 2013, Boston, MA, USA, low risk of bias (53)	*N =* 121	Families with 2–5-year-old children with a TV in child's bedroom. About 33% identified as Black American and 51% identified as Latinx.	Text messages plus coaching calls and home visits.	Bilingual health educators used MI techniques to review progress and setbacks to behavior change, discuss goals, and provide an activity or tool to support behavior change. Monthly coaching calls were designed to assess progress on making changes, provide support for challenges, and reinforce study messages. The intervention focused on promotion of the key household behaviors with particular attention to achieving the goals in low-resource home environments. Text message content focused on the adoption of household routines discussed during coaching calls and home visits.	Duration: 6-months. - Dose: Twice weekly text messages for the first 16 weeks and weekly text messages for last 8-weeks; Monthly coaching calls; Four home visits.	- Parents: Frequency of meals where at least some family members ate together in past 7 days. - Child: Sleep duration, screen time, BMI.
Newton et al., 2014, Louisiana, USA, high risk of bias (54)	*N =* 27 dyads	6–10-year-old children and one of their parents/guardians. About 59% identified as Black American.	Website and text messages.	Parent/guardian website provided access to view their child's daily step goal, monitor their child's step counts, view a color-coded steps/day graph to see how their child's daily steps compared to target step goal, and read weekly behavioral articles. Text messages were designed to help parents/guardians encourage their child's physical activity, remind parents/guardians of behavioral concepts presented in the website articles, and motivate parents/guardians to support their child's behavior change.	Duration: 12 weeks. Dose: Website updated weekly; about seven text messages/week in minimal intervention group; about 13 text messages/week in intensive intervention group.	- Parent/Guardian: Home and Neighborhood Food Environment Questionnaire (FFQ). - Child: Sedentary behavior, FFQ, pedometer, BMI, waist circumference, body fat percentage, fat free mass.
Nezami et al., 2018, Chapel Hill, NC, USA, low risk of bias (55)	*N =* 51 dyads	Parent/guardians (mothers) with overweight or obesity, who had a 2–5-year-old child that consumed ≥ 12 fluid oz./day of sugar sweetened beverages (SSB). About 44% identified as Black American.	Website, text messages, e-mail.	The goal of the intervention was to slowly reduce the child's SSB/juice consumption until the child was consuming 1 serving per day (1 child serving = 4fl. oz.). Parent/guardians self-monitored their weight, number of servings of caloric beverages, and the child's servings of SSB/juice in a paper or smartphone diary. Parent/guardians received a text message prompt each week to submit their diaries, which were used to create personalized feedback. Feedback was delivered via email and was tailored to whether specific goals had been met. Parents/guardians completed monthly brief questionnaires in which they selected their greatest barrier to meeting their goals. Reported barriers were used to provide additional personalized feedback in subsequent feedback email. Parents/guardians accessed lessons on a mobile website. The lessons focused on behavioral strategies to achieve goals, including parent/guardian-child communication, problem solving, skills targeted to maternal weight loss such as reading food labels and relapse prevention strategies. Text messages included link to lessons, tips, motivational messages, and goal progress assessments with a semi-automated tailored feedback message based on responses.	Duration: 6 months. Dose: One 75-min in-person group session; Website content updated weekly during weeks 2–12 and biweekly during weeks 13–24; 3–4 text messages/week; Weekly emails on self-monitoring for first 12-weeks then bi-weekly emails for next 12-weeks.	- Parent/Guardian: 24-h dietary recall, BMI. - Child: 24-h dietary recall.
Robinson et al., 2010, Oakland, CA, USA, low risk of bias (56)	*N =* 261	8–10-year-old Black American girls and their parents/guardians.	Videotaped feedback and TV allowance electronic time manager.	The after-school dance intervention included dance performances every 8 weeks for families and friends; videotaped feedback; opportunities for girls to teach each other and choreograph routines; opportunities for participant choice and control; and performances at public events. Sisters Taking Action to Reduce Television (START) was a home-based screen time reduction intervention designed to incorporate African or Black American history and culture to reduce screen time. Young Black American female mentors met with families in their homes to deliver each lesson.	Duration: After school dance intervention was 9-months; START was conducted over 2-years. Dose: After school dance intervention offered 5 days/week for 2.5 h; START 12–24 lessons over 2-years.	- Parents/Guardians: Black American cultural identity. - Child: Accelerometer, screen time, meals eaten with television on, 24-h dietary recall.
Taveras et al., 2017, Massachusetts, USA, low risk of bias (57)	*N =* 721	2–12-year-old children with a BMI ≥85th percentile from six primary care practices; about 33% identified as Black American and 21.8% as Latinx.	Text messages, email, video calls, and online community resource map.	Clinicians received a computerized, clinical decision support (CDS) alert during primary care visits identifying children with a BMI ≥ 85th percentile. They also received additional CDS tools to assist in overweight and obesity management of children. Clinicians provided parents/guardians with a set of educational materials to support behavior change. The materials focused decreasing screen time and SSBs; improving diet quality; increasing moderate and vigorous physical activity; and improving sleep duration and quality. In the enhanced primary care + coaching arm, parents/guardians received individualized coaching tailored to their socio-environmental context from health coaches who used MI techniques. Health coaches contacted parents/guardians by phone, videoconference, or in-person visits, according to parent/guardian preference. Parents/guardians received text messages or emails, following each coaching session with educational materials to support behavior change goals. At each contact, health coaches used an online community resource map to identify resources within each parent/guardian's community that could support behavior change. Parents/guardians also received a 1-month free YMCA membership and were invited to attend a healthy grocery shopping program.	Duration: 1 year. Dose: Video calls every other month; Twice weekly text messages or emails.	- Parent/Guardian: Parent Resource Empowerment Scale. - Child: BMI.
Trude et al., 2019, Baltimore, MD, USA, low risk of bias (58)	*N =* 533	Families with 9–15-year-old children residing in low-income, predominantly Black American neighborhoods with low access to healthy foods.	Text messages and social media.	The intervention used an ecological and food systems approach. Individual-level components were based in community recreation centers, using youth leaders to provide education and nutrition skills to youth. The family-level included social media and text messages to target family-level nutrition behaviors. Recipes, news, and intervention-specific activities were featured in social media and text messages. Text messages and social media platforms provided parents/guardians with guidance to set and achieve dietary goals for themselves and their families, as well as promoting intervention community activities. The intervention promoted healthy foods/beverages and behaviors in three sequential phases, each lasting two months: (1) healthier beverages, (2) healthier snacks, and (3) healthier cooking methods.	Duration: 6 months. Dose: Text messages sent three times/week.	- Parent/Guardian: Fruit and vegetable intake, household food preparation, frequency of food acquisition.
White et al., 2004, Louisiana, USA, unclear risk of bais (59)	*N =* 57	11–15-year-old Black American girls who were overweight and had a parent/guardian with obesity (BMI>30).	Website and email.	Participants were provided with a home computer and free internet access. Participants visited website weekly and accessed material which focused on weight loss, and included information on nutrition, physical activity, and healthy food choices. Behavior change strategies were highlighted in weekly emails sent by a weight management case manager. Topics included: self-monitoring, goal setting, problem solving, behavioral contracting, and relapse prevention. Participants completed daily food records and submitted them on the website. Food records were reviewed by a dietician. Automated feedback was also provided. A computer program generated an image of the Food Guide Pyramid and indicated the extent to which the food records complied with the recommended nutritional values.	Duration: 2-years (6-month outcomes reported). Dose: Website content updated, and emails sent weekly.	- Parent/Guardian: Body fat (DEXA), BMI. - Child: Body fat (DEXA), BMI, dietary self-efficacy, 24-h dietary recall, and FFQ.
Wright et al., 2013, Boston, MA, USA, low risk of bias (60)	*N =* 50 dyads	9–12-year-old children with obesity and their parents/guardians attending an urban pediatric outpatient clinic. The majority of participants (72%) identified as Black American.	Interactive voice technology (IVR).	The IVR monitored, educated, and counseled parents/guardians and children on healthy weight management and screen time. The IVR spoke using text-to-speech technology. Participants communicated by speaking or by pressing keys on telephone keypad. The IVR conversation was tailored to each participant; it asked questions and provided tailored feedback. Child intervention: Concepts from the Traffic Light Diet (TLD) and the Student Media Awareness to Reduce Television program guided the child IVR conversations (e.g., increase consumption of green foods, reduce TV time to < 2 h/day). Conversation objectives included: (1) learn the TLD; (2) learn about rules; (3) self-monitor diet and screen time behaviors; and (4) set up contracts and rewards. Parent/Guardian intervention: IVR conversation content mirror children's conversation to encourage support and teamwork. Conversation objectives: (1) create a healthy home; (2) role modeling; (3) developing respectful parent/guardian-child relationship; (4) using praise and encouragement to motivate children; (5) follow the TLD with child to support efforts. Children and parents/guardians were provided a guidebook to support the calls. Data captured in the child IVR system were sent to child's pediatrician via electronic health record. Recommendations for praising, encouraging and problem solving behaviors were provided to pediatrician.	Duration: 12 weeks Dose: Two calls per week.	- Parent/Guardian: Block Data Systems dietary screener, screen time. - Child: Block Data Systems dietary screener, screen time, and time spent on recreational activities.

Of the included studies, six used text messaging as part of their DHI (49, 53–55, 57, 58). Of these six studies, one was a text message-based only DHI and decreased the number of text messages sent to parents/caregivers over the study period from three text messages per week to 1–2 text messages sent per week (49). The remaining five studies utilized text messages in combination with another intervention strategy such as a website or e-mail communication (54, 55, 57, 58, 61).

Five of the 12 studies utilized a website as part of their DHI (50–52, 54, 55, 59) and only one of these studies was a website-only DHI (52). Most of these studies updated the website content on a weekly basis (*n* = 4) (50, 51, 54, 59), one study updated the website content on a weekly basis and then decreased this to a bi-weekly basis (55), while one study consisted of six, self-paced website modules (52).

Four studies used e-mail as part of their DHI strategy (50, 55, 57, 59). Two of these studies sent e-mails to parents/caregivers on a weekly basis (50, 59), one study sent e-mails on a weekly basis during the first half of the DHI and then on a bi-weekly basis during the second half of the DHI (55), and one study sent either text messages or e-mails twice weekly, depending on the parent's/caregiver's preference (57). All the studies that used e-mail as part of their DHI strategy did so in combination with other DHI strategies.

Other DHI strategies utilized include Interactive Voice Technology (IVR) calls that occurred twice weekly (60), video stories updated weekly on a website (51), monthly coaching calls and home visits (53), video feedback provided at an after-school dance program offered 5 days/week and a TV time manager provided to families (56), an online community resource map provided to parents/caregivers after bi-monthly video calls (57), and social media (58).

### Parent/Caregiver Participation

Parent/Caregiver participation measures and outcomes, and retention/loss to follow-up rates are reported in Table 2. Measurement of parent/caregiver participation in the DHIs included in this review varied. Two of the studies assessed text message response rate (49, 55) and four of the studies assessed the overall number of text messages sent (49, 54, 57, 58). Less than half of the studies (*n* = 5) assessed the average number of times parents/caregivers logged into the DHI website (50–52, 54, 59) while half of the included studies (*n* = 6) assessed parent/caregiver satisfaction with the DHI (49, 51, 53, 55, 57, 60). Other parent/caregiver participation measures included feasibility of IVR calls (60), number of dance lessons attended and number of TV time managers connected (56), number of completed video calls (57), an Intervention Exposure Questionnaire (IEQ) (58), and the number of completed weekly quizzes and self-monitoring forms submitted (59).

**Table 2:** Parent/caregiver participation in digital health interventions and behavioral and weight status outcomes.

**Study**	**Parent/Caregiver participation measures**	**Parent/Caregiver participation outcomes**	**Retention/Loss to follow-up/Withdrawal Rate**	**Parent/Caregiver behavioral and/or weight status outcomes**	**Child behavioral and/or weight status outcomes**
Armstrong et al. (49)	- Text message response rate. - Acceptability of text message frequency, timing, and content. - Perceived usefulness. - Text message dose.	Text message response rate: - Parents/caregivers responded at least once to 80% of text messages and parents/caregivers responded twice or more to 30% of text messages. Acceptability: - 81% of parents/caregivers enjoyed receiving messages; 92% felt they were personalized; 62% wanted to receive texts past the study period; and 92% would recommend to a friend. Perceived usefulness: - 95% of parents/caregivers perceived the frequency “just right,” and 95% said messages “almost always” or “always” helped them make a good decision about their child's health. Text message dose: - Participants received a mean of 60 messages over the study period.	−81% retention rate	- No significant differences observed.	- No significant differences observed.
Baranowski et al. (50)	Weekly log-on rates.	Weekly log-on rate: - Mean log-on rate for parents/caregivers was 47%.	- Reports camp attendance.	- Not applicable.	- No significant differences observed.
Cullen et al. (51)	Website log-on. Website evaluation: parents/caregivers asked to grade the program.	Website log-on: - Website log-on rate over intervention period was 86%. - 66% of parents/caregivers logged onto all 8 sessions. Website evaluation: - Parents/caregivers in both conditions reported liking the program components; 63 parents/caregivers graded it an A or B.	−66% of intervention families and 74% of control families completed all data collection surveys.	- Meat modification was significantly higher at follow-up for both intervention and control parents/caregivers. - The reduced-fat scale and the substitutions scale was significantly higher at post-intervention and at follow-up for intervention parents/caregivers. - The fruit and vegetables scale was significantly higher at post-intervention and follow-up for intervention parents/caregivers and at follow-up for control group parents/caregivers.	- No significant differences observed.
Frenn et al. (52)	Feasibility: Number of returned completed consent forms, and visits to intervention components in the online program.	Feasibility - Of the 161 parents/caregivers invited, no response was received from 98. - Parents/caregivers of 5th graders = 52% response rate. - Parents of 7th−8th graders = 30% response rate at public school; 36% at private school. - 81% of parents/caregivers and children completed pretest data. - 9 parents/caregivers who agreed to complete the online modules did not.	−30% retention for parents/caregivers - 90% for children	- No significant differences observed.	- No significant differences observed.
Haines et al. (53)	Parent/caregiver satisfaction with program.	−89% of parents/caregivers reported being “satisfied” or “very satisfied” with the program. - 98% were “satisfied” or “very satisfied” with the counseling received during home visits. - 98% were “satisfied” or “very satisfied” with the counseling received during coaching calls. - 98% of parents reported they would recommend the program to friends and family.	- Intervention group = 6 lost to follow-up. - Control group = 2 lost to follow-up.	- No significant differences observed.	- Significant decrease in BMI by a mean of 0.18 in the intervention group and increased by 0.21 in the control group at 6-mos. - Significant increase in sleep duration by 0.56 h/day in the intervention group and decreased by 0.19 h/day in the control group. - Significant, larger decreases in weekend TV viewing were observed among intervention group compared with the control (−1.06 h/d; 95% CI, −1.97 to −0.15).
Newton et al. (54)	- Website log-on rates and views. - Self-monitoring of step counts. - Text message response frequency.	- Website log-on rates and views: - 38% of parents/caregivers accessed 9 or more articles; 23% accessed between 4 and 8; 38% accessed < 4 articles; 2 parents never accessed an article. - Parents/caregivers accessed 70% of articles in Month 1; 60% in Month 2; and 37.5% in Month 3. Self-monitoring of step counts: - Parents/caregivers visited the steps/day graph an average of 25.3 (SD 24.5) times over the course of the study (2.1 times/week). Number of text messages sent: - Parents/caregivers in control group sent 162 (0.96/week) text messages. - Parents/caregivers in intervention group sent 419 (2.7/week) text messages.	- None lost to follow-up.	- No significant differences observed.	- For pedometer step counts, children in both groups demonstrated significant increases in steps by 1427.6 (SD 583.0) for control and 2832.8 (SD 604.9) for intervention. The between-group and group by time difference was not statistically significant. - No other significant differences detected.
Nezami et al. (55)	- Program utilization. - Program satisfaction.	Program utilization:- Parents/caregivers submitted an average of 21.5 (4.3) out of 24 weeks of self-monitoring texts and responded to an average of 15.4 (1.7) out of 18 goal progress assessment texts.	- Retention rate: 86% at 3-months and 82% at 6-months.	- Self-monitoring and goal progress assessment texts predicted greater weight loss; intervention group lost 2.4 kg, which was significantly greater than the weight gain of 0.9 kg observed among control group.	- Significant difference observed for change in child SSB/juice intake at both 3 and 6 months; children in intervention group had a greater reduction compared with the control group at 3 months (−9.9fl. oz. day vs. −2.7fl. oz. day) and 6 months (−9.7fl. oz./day vs. −1.7fl. oz./day).
		- Parents/caregivers reported spending ~50 min/week completing study-related activities. - Program satisfaction - All intervention parents/caregivers reported that they would “probably” or “definitely” recommend the program to a friend. - 91% of parents/caregivers reported being satisfied with the program.		- A greater proportion of intervention parents/caregivers (37%) reached a weight loss of 3% compared to control (4%), and a greater proportion reached a weight loss of 5% compared to control (22 vs. 0%). - Intervention parents/caregivers had a greater reduction in caloric beverages compared with the control parents/caregivers (−11.5fl. oz./day vs. 0.4fl.oz./day).	- Significant difference observed for meeting SSB goal, 52% of children met the goal of consuming <4 oz./day of SSB/juice at 6 months, compared with 21% in the control group.
Robinson et al. (56)	- Number of START lessons received. - Use of TV Allowance time manager.	START lessons: - Delivered mean of 12.4 out of 25 START lessons. - 70% of families received at least the first 7 lessons, 29% received 7–14 lessons, 34% received 15–20 lessons, and 7% received 21 or more. TV Allowance: - 77% of families hooked up at least one TV allowance manager (12% two or more). - The mean reported weekly screen time budget goal was 10.0 ± 2.4 h.	−18 girls were lost to follow-up; 94% of girls in the intervention condition and 92.1% of girls in the control condition completed at least one follow-up assessment.	- Treatment parents had significantly increased preference for Black American things compared to control parents.	- No significant differences observed.
Taveras et al. (57)	- Text messages: Percent received and satisfaction. - Neighborhood Resource Guide: Percent received and satisfaction. - Percent completion of health coach visits.	Text messages: - In the enhanced primary care group, 91% of parents/caregivers reported they received text messages and 53% were satisfied with their content. - In the enhanced primary care + coaching group, 100% of parents/caregivers reported receiving the study text messages and 72% were very satisfied with their content. - Neighborhood Resource Guide - In the enhanced primary care group, 60% of parents/caregivers reported receiving the Neighborhood Resource Guide and 66% reported being very satisfied with its content. - In the enhanced primary care + coaching group 96% reported receiving neighborhood resource information and 76% were very satisfied with the information. Health Coach Visits: - In the enhanced primary care, 65% completed all 6 visits with a health coach.	Retention rate for intervention group: - 90% for parents/caregivers - 93% for children.	- No significant differences observed.	- No significant differences observed.
Trude et al. (58)	Intervention Exposure Questionnaire: Self-reported viewing of communication materials, participation in food environment intervention activities, enrollment in social media, receipt of text messages.	- Parents/caregivers presented an overall exposure score of 1.38 points, SD ± 1.2 (range: 0–6.9). - The Communication Materials exposure score was 0.6 points. - The Food Environment exposure score was 0.3 points. - The Social Media exposure score was 0.2 points. - Text Messaging exposure score (based on the frequency of text messages received per week) was 1.10 points.	- Attrition rate = 24.9%	- No significant differences observed for food acquisition, home food preparation, and daily consumption of FV. - For each one-point increase in exposure score, there was a 0.24 increase in mean daily fruit serving for parents/caregivers in intervention group (0.24 ± 0.11; 95% CI 0.04; 0.47). - For each one-point increase in the social media exposure score, there was an increased three servings of daily fruit intake (3.16 ± 0.92; 95% CI 1.33; 4.99) and an increase in daily fruit and vegetable intake (2.94 ± 1.01; 95% CI 0.96; 4.93). - Higher social media exposure score was associated with increased unhealthful food acquisition score (0.47 ± 0.23; 95% CI 0.02; 0.93).	- Not reported.
White et al. (59)	- Website log-on rates. - Weekly quiz completion. Frequency counts of the number of food diaries and exercise self-monitoring forms submitted.	- Website log-on rates - Intervention group website visits: mean of 557.3 (SD 500.4). - Control group website visits: mean of 226.8 (SD 161.8). - Other parent participation outcomes not reported.	Lost to follow-up: 17.8% in the intervention group and 6.9% in the control group.	No significant differences detected.	Adolescents in the behavioral group lost more fat than those in the control group (*F* = 3.44, *p* < 0.05, *b* = 0.28, *p* < 0.05).
Wright et al. (60)	- Use, credibility, and satisfaction. - Feasibility.	Use, credibility, and satisfaction: - Of parents/caregivers who made ≥ 1 call, ≥75% agreed it was useful, easy to use, made for people like them, credible, and helped them eat healthy foods, and watch less TV. - 100% of parents/caregivers would recommend it to a friend, and 100% agreed they liked it because they could use it at home. Feasibility: - 76% of parents/caregivers called the IVR at least once; of those who called at least once, the mean number of total calls was 9.1 (SD = 5.2). - Parents/caregivers made an average of 5.2 (SD = 2.8) education and behavior calls and 3.9 (SD = 2.6) tracking calls.	Lost to follow-up: 12.5% in the intervention group and 15.4% in the control group.	- Intervention parents/caregivers consumed 1.1 more cups of fruit per day than control [*F*_(1,40)_ = 4.22, *p* = 0.046); but intervention parents/caregivers consumed fewer servings of vegetables than control parents/caregivers [*F*_(1,40)_ = 6.88, *p* = 0.012]. - Analyses of high vs. low users of IVR found that the high users consumed significantly fewer calories compared to the low users.	No significant differences observed.

Level of parent/caregiver participation varied. Overall, in studies that included text messaging response rate was high (above 80%) and text message dose was close to as intended (49, 54, 55, 57, 58). There was also moderate to high satisfaction with the DHI among parents/caregivers (49, 53, 55, 57). In studies with a website component, the mean website logon rate in two of the studies ranged from 47 to 86% over the 2-month DHI period (50, 51) while one study reported that the mean number of times logged onto the website was 557 times over the 6-month DHI period (59). One study reported that the mean number of times parents/caregivers logged onto the website decreased over the duration of the 12-week DHI (54). Other parent/caregiver participation outcomes reported by studies with a website component were high satisfaction with the DHI (51), frequent logons to view their child's self-monitoring of behavior progress (54), and high completion rate of online modules (52).

In the IVR DHI, there was high satisfaction with IVR calls and 76% of parents/caregivers completed at least one IVR call (60). For those participating in the DHI with dance lessons plus videotaped feedback, 70% attended at least the first seven lessons and 77% of parents/caregivers connected their family TV to the time manager (56). Lastly, for the study with the social media component, overall social media exposure was low (mean social media exposure score: 0.2 (observed range: 0.0–2), possible highest score: 2) (58). No parent/caregiver participation outcomes were reported for email strategies.

### Parent/Caregiver and Child Behavioral Outcomes

Parent/Caregiver and child behavioral and weight status outcomes are also reported in Table 2. Of the studies included in this review, half (*n* = 6) reported non-significant outcomes for parents/caregivers and/or children in terms of behavioral and/or weight status outcomes for those in the intervention condition compared to the control (49, 50, 52, 54, 56, 57).

Four found significant positive changes in the dietary (51, 55, 58, 60) and weight status outcomes (55) of parents/caregivers in the intervention condition compared to those in the control. One of the included studies saw positive changes in the dietary outcomes of children in the intervention condition compared to children in the control condition (55), and two of the studies saw significant, positive changes in the weight status outcomes of children in the intervention condition when compared to the control (53, 59).

Of the studies with reported significant changes in the behavioral and/or weight status outcomes for parents/caregivers and/or children (*n* = 6), three reported high DHI utilization (51, 55, 59, 60) and three studies reported high satisfaction with the DHI (51, 53, 55). One of the studies with reported significant changes in behavioral outcomes reported that each one-point increase in the DHI exposure score was associated with daily fruit intake, and despite the low exposure to the social media component, for each one-point increase in the social media exposure score, there was an increase in daily fruit intake and an increase in unhealthful food acquisition (58). Lastly, in studies with reported non-significant changes in the behavioral and/or weight status outcomes for parents/caregivers and/or children (*n* = 6), parent/caregiver participation ranged from low to high.

## Discussion

The primary aim of this systematic review was to identify Black American and/or Latinx parents'/caregivers' level of participation in DHIs for obesity prevention or treatment for their children. The secondary aim was to determine the relationship between parent/caregiver participation and behavioral, specifically diet and physical activity, and weight status outcomes for both parents/caregivers and children. Regarding parent/caregiver participation, across most DHI studies included, participation was relatively high, apart from a study that included a social media-based component; participation, as measured by an exposure score, for the social media-based component was low. However, this study did find that an increase in social media exposure score was associated with both positive and negative dietary behavior outcomes. These parent/caregiver participation findings offer promising support for the feasibility of DHIs for obesity prevention and treatment interventions in Black American and Latinx families with young children (52). However, only half (*n* = 6) of the included studies reported significant improvements in obesity or related health behaviors for parents/caregivers and/or their children. Of these 6 studies, three of them reported high parental/caregiver participation rates and 3 reported high satisfaction rates. These findings suggest that participation and satisfaction may have an impact on health behavior change and weight status, but the small number of studies suggests that additional research needs to be conducted to determine whether these engagement factors or other factors predict responsiveness to the DHI.

### Implications and Recommendations for Future Research

Given the range of DHI strategies used and the varied measures of parent/caregiver participation utilized within the studies, it is difficult to compare the studies in terms of parent/caregiver participation and how their participation may vary by DHI strategy or how their participation may influence behavioral and health outcomes. More consistent measurement of participation outcomes is needed across DHI studies and researchers need to adequately plan for and collect participation data so that we can better understand the relationship between parent/caregiver participation, DHI strategies, and behavioral and health outcomes. Due to technological advancements, it is now possible to collect data on both participation with a DHI's features, as well as participation in specific behavior change DHI strategies (62). Given this, there is an opportunity for researchers to track and assess participation for their duration of their study and to collect DHI participation data similarly to how others are, which would make comparisons across studies more feasible. Having such information would assist in identifying the optimal dose and intensity of DHI intervention activities to achieve desired behavioral and health outcomes (58). Additionally, such data would allow researchers to follow the advancements in DHI research over time, and provide researchers the opportunity to assess the benefits and limitations of various types of behavioral-based DHI strategies in terms of impact and outcomes (34). Lastly, it would also be useful for researchers to collect qualitative data from parents/caregivers and other stakeholders (e.g., think-aloud interviews, key informant interviews) to better understand and explain experiences with the DHI (63). Such approaches are consistent with community engagement and human-centered design approaches, which aims to design interventions with the user in mind, which is particularly important in health equity research (64, 65).

Other efforts need to focus on increasing our understanding of how best to reach Black American and Latinx families to enroll in DHI research and how best to maintain their engagement in the DHI. One way to increase our understanding of this is to understand how participation in a DHI may be affected by contextual factors (66, 67). DHIs are typically delivered in real-world settings, where everyday health behavior change may or may not occur (68). This means that DHI participation may be positively or negatively affected by contextual influences such as family, school, work, or broader community and societal influences (69). DHI participation may also be affected by parent's/caregiver's ability to access internet services. Black Americans and Latinx adults are almost twice as likely as non-Latinx white adults to have canceled internet services because of the expense and are more likely to access internet services in community venues such as libraries (33). Contextual data can be collected in a multitude of ways. For example, ambient or mobile device sensors to capture data such as location, weather, or busyness of day based on calendar, structured or semi-structured interviews, observational data, ecological momentary assessments, or contextual assessments of various settings (e.g., home, neighborhood, food environments) (69–71). None of the studies included in this review reported on contextual factors. Such data would allow researchers to better understand how contextual factors may impact one's participation in a DHI so that the DHI can be delivered at times parents/caregivers are more likely to engage with the DHI (e.g., text messages sent at specific times of day or days of the week), or more likely to engage in the desired behavior, or so that it can incorporate features that may address contextual factors to further support behavior change. Strategies such as this are commonly used in digital just-in-time adaptive interventions that target individuals at suitable moments, particularly when they have the opportunity to engage in a healthy behavior and are more receptive to support offered by the DHI (72). But to target individuals at these opportune moments, contextual data is required.

DHIs are just one strategy to advance healthy eating and physical activity and support diverse parents in modeling healthful behavior. As technology advances these types of interventions will become more powerful. However, dietary and physical activity behaviors are complex and are influenced by multifactorial determinants beyond the individual-level (73). Not one specific type of individual-level intervention can stem the tide of obesity inequities until we address and target multilevel determinants from the individual-, the family-, the environmental-, and the policy-levels (74). Multilevel interventions are one potential solution to address this but are often costly. Team science is an emerging area of exploratory and intervention public health research that promotes interdisciplinary and transdisciplinary collaboration to address public health phenomena (75). Multifactorial causes of public health phenomena such as obesity inequities requires greater collaboration among scientists trained in different fields (76–78). With a team science and transdisciplinary approach, researchers can work jointly to synthesize, apply, and extend their discipline-specific theories, concepts, and/or methods to better incite discovery and inform solutions to reduce obesity inequities (74). Therefore, it is recommended that team science approaches and partnerships are utilized to address the obesity inequities faced by Black American and Latinx families. This can include transdisciplinary partnerships (e.g., experts from public health, psychology, computer science, policy, nutrition, kinesiology, among others) in addition to academic-industry partnerships or academic-community partnerships. Our recommendations for future research are summarized in Table 3.

**Table 3:** Recommendations for future DHI research.

**DHI study component or consideration**	**Proposed recommendation**
Parent/caregiver participation measurement	- If utilizing multiple DHI strategies or features, collect data on participation from each of the DHI features. - Collect data on participation from each of the DHI behavior change strategies utilized. - Track and evaluate participation throughout DHI study to identify optimal dose and intensity. - Utilize participation measures from previous studies so that participation can be compared across DHI studies. - Collect qualitative data to better understand experiences with the DHI.
Parent/caregiver reach and participation	- Report recruitment rates and strategies for researchers to replicate. - Collect contextual data to understand how best to reach and engage Black American and Latinx parents in DHI (e.g., ambient or mobile device sensors, qualitative data, observational data, ecological momentary assessments, contextual assessments of various settings where DHI may be used by parents/caregivers). - Subgroup analyses to determine if race/ethnicity is a moderating factor in treatment effects or participation.
Team science-based approaches	- Development of transdisciplinary teams (e.g., experts from public health, computer science, artificial intelligence, behavioral science, nutrition, kinesiology, policy, among others), in addition to academic-community and academic-industry partnerships, to best synthesize, apply, and extend discipline-specific theories, concepts, and/or methods to better develop and implement DHIs for obesity prevention and treatment.

### Strengths and Limitations

Our study should be considered within the context of several limitations. First, only 12 studies met our inclusion criteria limiting the amount of data available to make meaningful conclusions. Second, given our focus on a process-related outcome, parent/caregiver participation, which does not influence how the primary outcomes were assessed, we did not assess studies for quality or bias. This limits our ability to conduct a formal assessment of the quality of evidence provided (79). Third, although we conclude that parent/caregiver participation was moderate to high across most of the studies, not all studies reported on recruitment rates. Previous research has found that recruiting Black American and Latinx participants in research studies is a challenge (80, 81) and although we were able to capture studies that successfully recruited samples that were at least 25% Black American and Latinx, these participants may have been highly motivated to engage given that they participated in the studies. Future research should determine predictors of and barriers to enrolling in DHIs for obesity prevention and treatment within these populations to increase enrollment in future studies. Further, there are some limitations of the literature overall that are noteworthy. First, the variation in types of digital interventions and intervention components complicates the broad conclusions we can make about which intervention components are the best in terms of maintaining participation and changing diet and physical activity behaviors. Related, the time of intervention and dose of the intervention varied across studies so it is difficult to determine with certainty which components and how intense the intervention should be to result in meaningful change.

This is the first systematic review of Black American and Latinx parent/caregiver participation in obesity prevention and treatment DHIs for their children. This review is an important step in increasing our understanding of engaging two populations that are systematically and disproportionately affected by overweight and obesity in the United States. Additionally, this review focused on randomized controlled trials, which are considered the gold standard study design. Lastly, a team of reviewers were involved during the selection and data extraction process which minimized the potential for selection bias.

## Conclusions

Given these limitations, future research should clearly define recruitment rates and recruitment strategies so that other researchers can replicate methods that are successful. Additionally, it is important to determine which treatment components and what intensity is necessary for meaningful changes. Further, it is important to test longitudinal changes in health behaviors and obesity-related outcomes from DHIs to determine whether the changes sustain over time. Finally, future research should determine predictors of responsiveness to the intervention (e.g., parent/caregiver participation, satisfaction, etc.) and, if a study includes more than one race/ethnicity within its sample, subgroup analyses should be conducted to determine if DHI treatment effects or participation are moderated by race/ethnicity.

Overall, our study represents an important first step to determining parent/caregiver participation and behavior change outcomes for DHIs in two populations systematically and disproportionately impacted by obesity; Black American and Latinx families. Our results lay the groundwork for the developing and testing of future DHI interventions with the explicit goal of increasing Black American and Latinx parental/caregiver participation and considers the need to expand our DHI research methodologies to better address obesity inequities among diverse families.

## Data Availability Statement

The original contributions presented in the study are included in the article/Supplementary Material, further inquiries can be directed to the corresponding author/s.

## Author Contributions

JS-F and MF conceptualized and designed the study. JS-F, AO'D, MC, and JB conducted the screening of titles, abstracts, and full-text articles and extracted the data of each study. All authors contributed to the drafting of the manuscript and approved of the final version.

## Conflict of Interest

The authors declare that the research was conducted in the absence of any commercial or financial relationships that could be construed as a potential conflict of interest.
