# Supplementary material for: Black American and Latinx Parent/Caregiver Participation in Digital Health Obesity Interventions for Children: A Systematic Review
Source: Front Digit Health. 2021 Jun 15;3:687648. doi: 10.3389/fdgth.2021.687648 (PMC8522024; doi:10.3389/fdgth.2021.687648)
Supplement: Supplementary file 1 [file Data_Sheet_1.docx]

Supplementary Material

# Supplementary Data

## Search strategy

### ERIC

#### Obesity

TI(obes* OR BMI OR "body mass index" OR "obesity prevention" OR "body weight" OR overweight OR "weight status" OR "body fat" OR "prevent obesity" OR "healthy weight" OR adipos* OR "waist circumference" OR "body weight changes" OR anthropometry OR "over weight" diet OR "healthy eating" OR "energy density" OR "feeding behavior" OR "dietary habit" OR snack* OR "mealtime environment" OR meal* OR food OR "food habit" OR water OR "healthy snack" OR nutrition OR nutrient OR vegetable* OR fruit* OR juice OR "meal time behavior" OR "eating behavior" OR "food quality" OR "food preferences" OR "nutrition education" OR "nutrition quality" OR "dietary intake" OR consumption OR "whole grain" OR "sugar sweetened beverage" OR "eating habit" OR "improved access" OR availability OR intake OR "healthy eating habit" OR "healthy food" OR "improved consumption" OR "improved intake" OR "dietary quality" OR dietary OR "food and beverage consumption" "physical activity" OR active OR movement OR "active play" OR recess OR "play time" OR playtime OR "motor skill" OR sedentary OR exercise OR play OR "free play" OR "physical fitness" OR "motor activity" OR recreation OR "physical activities" OR "sedentary behavior") OR

AB(obes* OR BMI OR "body mass index" OR "obesity prevention" OR "body weight" OR overweight OR "weight status" OR "body fat" OR "prevent obesity" OR "healthy weight" OR adipos* OR "waist circumference" OR "body weight changes" OR anthropometry OR "over weight" diet OR "healthy eating" OR "energy density" OR "feeding behavior" OR "dietary habit" OR snack* OR "mealtime environment" OR meal* OR food OR "food habit" OR water OR "healthy snack" OR nutrition OR nutrient OR vegetable* OR fruit* OR juice OR "meal time behavior" OR "eating behavior" OR "food quality" OR "food preferences" OR "nutrition education" OR "nutrition quality" OR "dietary intake" OR consumption OR "whole grain" OR "sugar sweetened beverage" OR "eating habit" OR "improved access" OR availability OR intake OR "healthy eating habit" OR "healthy food" OR "improved consumption" OR "improved intake" OR "dietary quality" OR dietary OR "food and beverage consumption" "physical activity" OR active OR movement OR "active play" OR recess OR "play time" OR playtime OR "motor skill" OR sedentary OR exercise OR play OR "free play" OR "physical fitness" OR "motor activity" OR recreation OR "physical activities" OR "sedentary behavior") OR

DE (obesity OR "body composition" OR "body weight" OR "child health" "eating habits" OR "food" OR "nutrition" OR "food service" "physical fitness" OR "physical activity level" OR "physical activities" OR "exercise" OR "physical education")

#### Digital health

TI("digital health" OR mhealth OR ehealth OR "m-health" OR "e-health" OR text OR "text-based" OR internet OR computer OR computers OR SMS OR iphone OR iphones OR phone OR phones OR smartphone OR smartphones OR mobile OR tablet OR tablets OR "video game" OR "video games" OR videogame OR videogames OR technology OR technologies OR web OR online OR "social media" OR "social network" OR "social networks" OR email OR "e-mail" OR elearning OR "e-learning" OR telehealth OR app OR "mobile app" OR "mobile apps" OR applications OR "mobile application" OR "mobile applications" OR "mobile device" OR "mobile devices" OR "web based" OR "web-based" OR "cell phone" OR "cell phones" OR cellphone OR cellphones OR "smart phone" OR "smart phones")

AB("digital health" OR mhealth OR ehealth OR "m-health" OR "e-health" OR text OR "text-based" OR internet OR computer OR computers OR SMS OR iphone OR iphones OR phone OR phones OR smartphone OR smartphones OR mobile OR tablet OR tablets OR "video game" OR "video games" OR videogame OR videogames OR technology OR technologies OR web OR online OR "social media" OR "social network" OR "social networks" OR email OR "e-mail" OR elearning OR "e-learning" OR telehealth OR app OR "mobile app" OR "mobile apps" OR applications OR "mobile application" OR "mobile applications" OR "mobile device" OR "mobile devices" OR "web based" OR "web-based" OR "cell phone" OR "cell phones" OR cellphone OR cellphones OR "smart phone" OR "smart phones") OR

DE("computer applications" OR "online therapy" OR "mobile phones" OR "internet" OR "telemedicine" OR "computer assisted therapy" OR "electronic health services" OR "mobile health" OR "social media" OR "online social networks" OR "websites" OR "mobile phones" OR "text messaging" OR "smartphones")

#### Parents/caregivers

TI(parent OR parents OR mother OR mothers OR father OR fathers OR guardian OR guardians OR caregiver OR caregivers OR parenting OR parental OR maternal OR paternal) OR

AB(parent OR parents OR mother OR mothers OR father OR fathers OR guardian OR guardians OR caregiver OR caregivers OR parenting OR parental OR maternal OR paternal) OR

DE("caregivers" OR "parenting" OR "parenting style" OR "parent child relations" OR "parent child communication" OR "fathers" OR "mothers")

#### Race/ethnicity

TI(Latinx OR Latino OR Latinos OR Latina OR Latinas OR Hispanic OR Hispanics OR "African American" OR "African Americans" OR "Black" OR "Race" OR "Racial" OR "Ethnicity" OR "Ethnicities" OR "Minority" OR "Minorities" OR "minority health") OR

AB(Latinx OR Latino OR Latinos OR Latina OR Latinas OR Hispanic OR Hispanics OR "African American" OR "African Americans" OR Black OR Race OR Racial OR Ethnicity OR Ethnicities OR Minority OR Minorities OR "minority health") OR

DE("Latinos/Latinas" OR "Blacks" OR "racial and ethnic differences" OR "racial and ethnic groups")

### PyscINFO

#### Obesity

MAINSUBJECT.EXACT.EXPLODE("body mass index") OR MAINSUBJECT.EXACT.EXPLODE("obesity") OR MAINSUBJECT.EXACT.EXPLODE("body fat") OR MAINSUBJECT.EXACT.EXPLODE("body weight") OR MAINSUBJECT.EXACT.EXPLODE("eating behavior") OR MAINSUBJECT.EXACT.EXPLODE("sugars") OR MAINSUBJECT.EXACT.EXPLODE("food") OR MAINSUBJECT.EXACT.EXPLODE("active living") OR MAINSUBJECT.EXACT.EXPLODE("physical activity") OR MAINSUBJECT.EXACT.EXPLODE("sedentary behavior") OR MAINSUBJECT.EXACT.EXPLODE("exercise") OR MAINSUBJECT.EXACT.EXPLODE("activity level") OR MAINSUBJECT.EXACT.EXPLODE("food intake") OR AB,TI("obes*") OR AB,TI("body mass index") OR AB,TI("obesity prevention") OR AB,TI("body weight") OR AB,TI("overweight") OR AB,TI("weight status") OR AB,TI("body fat") OR AB,TI("prevent obesity") OR AB,TI("healthy weight") OR AB,TI("adipos*") OR AB,TI("waist circumference") OR AB,TI("body weight changes") OR AB,TI("anthropometry") OR AB,TI("over weight")

#### Digital health

MAINSUBJECT.EXACT.EXPLODE("computer applications") OR MAINSUBJECT.EXACT.EXPLODE("online therapy") OR MAINSUBJECT.EXACT.EXPLODE("mobile phones") OR MAINSUBJECT.EXACT.EXPLODE("internet") OR MAINSUBJECT.EXACT.EXPLODE("telemedicine") OR MAINSUBJECT.EXACT.EXPLODE("computer assisted therapy") OR MAINSUBJECT.EXACT.EXPLODE("electronic health services") OR MAINSUBJECT.EXACT.EXPLODE("mobile health") OR MAINSUBJECT.EXACT.EXPLODE("social media") OR MAINSUBJECT.EXACT.EXPLODE("online social networks") OR MAINSUBJECT.EXACT.EXPLODE("websites") OR MAINSUBJECT.EXACT.EXPLODE("mobile phones") OR MAINSUBJECT.EXACT.EXPLODE("text messaging") OR MAINSUBJECT.EXACT.EXPLODE("smartphones") OR AB,TI("digital health") OR AB,TI("mhealth") OR AB,TI("ehealth") OR AB,TI("m-health") OR AB,TI("e-health") OR AB,TI("text") OR AB,TI("text-based") OR AB,TI("internet") OR AB,TI("computer") OR AB,TI("computers") OR AB,TI("SMS") OR AB,TI("iphone") OR AB,TI("iphones") OR AB,TI("phone") OR AB,TI("phones") OR AB,TI("smartphone") OR AB,TI("smartphones") OR AB,TI("mobile") OR AB,TI("tablet") OR AB,TI("tablets") OR AB,TI("video game") OR AB,TI("video games") OR AB,TI("videogame") OR AB,TI("videogames") OR AB,TI("technology") OR AB,TI("technologies") OR AB,TI("web") OR AB,TI("online") OR AB,TI("social media") OR AB,TI("social network") OR AB,TI("social networks") OR AB,TI("email") OR AB,TI("e-mail") OR AB,TI("elearning") OR AB,TI("e-learning") OR AB,TI("telehealth") OR AB,TI("app") OR AB,TI("mobile app") OR AB,TI("mobile apps") OR AB,TI("applications") OR AB,TI("mobile application") OR AB,TI("mobile applications") OR AB,TI("mobile device") OR AB,TI("mobile devices") OR AB,TI("web based") OR AB,TI("web-based") OR AB,TI("cell phone") OR AB,TI("cell phones") OR AB,TI("cellphone") OR AB,TI("cellphones") OR AB,TI("smart phone") OR AB,TI("smart phones")

#### Parents/caregivers

MAINSUBJECT.EXACT.EXPLODE("caregivers") OR MAINSUBJECT.EXACT.EXPLODE("parenting") OR MAINSUBJECT.EXACT.EXPLODE("parenting style") OR MAINSUBJECT.EXACT.EXPLODE("parent child relations") OR MAINSUBJECT.EXACT.EXPLODE("parent child communication") OR MAINSUBJECT.EXACT.EXPLODE("fathers") OR MAINSUBJECT.EXACT.EXPLODE("mothers") OR AB,TI("parent") OR AB,TI("parents") OR AB,TI("mother") OR AB,TI("mothers") OR AB,TI("father") OR AB,TI("fathers") OR AB,TI("guardian") OR AB,TI("guardians") OR AB,TI("caregiver") OR AB,TI("caregivers") OR AB,TI("parenting") OR AB,TI("parental") OR AB,TI("maternal") OR AB,TI("paternal")

#### Race/ethnicity

MAINSUBJECT.EXACT.EXPLODE("racial and ethnic differences") OR MAINSUBJECT.EXACT.EXPLODE("racial and ethnic groups") OR AB,TI("Latinx") OR AB,TI("Latino") OR AB,TI("Latinos") OR AB,TI("Latina") OR AB,TI("Latinas") OR AB,TI("Hispanic") OR AB,TI("Hispanics") OR AB,TI("African American") OR AB,TI("African Americans") OR AB,TI("Black") OR AB,TI("Race") OR AB,TI("Racial") OR AB,TI("Ethnicity") OR AB,TI("Ethnicities") OR AB,TI("Minority") OR AB,TI("Minorities") OR AB,TI("minority health")

#### Randomized controlled trials

SU.EXACT("Treatment Effectiveness Evaluation") OR SU.EXACT.EXPLODE("Treatment Outcomes") OR SU.EXACT("Placebo") OR SU.EXACT("Followup Studies") OR placebo* OR random* OR "comparative stud*" OR clinical NEAR/3 trial* OR research NEAR/3 design OR evaluat* NEAR/3 stud* OR prospectiv* NEAR/3 stud* OR (singl* OR doubl* OR trebl* OR tripl*) NEAR/3 (blind* OR mask*)

### PubMed

#### Obesity

"body size"[mesh] OR "anthropometry"[mesh] OR "body mass index"[mesh] OR "diet, food, and nutrition"[mesh] OR "beverages"[mesh] OR "nutrition policy"[mesh] OR "diet"[mesh] OR "exercise"[mesh] OR "obesity"[mesh] OR "adiposity"[mesh] OR "overnutrition"[mesh] OR "overweight"[mesh] OR "obesity"[tiab] OR "obese"[tiab] OR "overweight"[tiab] OR "diet"[tiab] OR "dietary"[tiab] OR "nutrition"[tiab] OR "adiposity"[tiab] OR "overnutrition"[tiab] OR "health promotion"[tiab] OR "physical activity"[tiab] OR "physical inactivity"[tiab] OR "exercise"[tiab] OR "exercising"[tiab] OR "BMI"[tiab] OR "body mass index"[tiab] OR "childhood obesity"[tiab] OR "sedentary behavior"[tiab]

#### Digital health

"internet"[mesh] OR "mobile applications"[mesh] OR "wearable electronic devices"[mesh] OR "communications media"[mesh] OR "telemedicine"[mesh] OR "social networking"[mesh] OR "computers"[mesh] OR "software"[mesh] OR "cell phone"[mesh] OR "digital health"[tiab] OR "mhealth"[tiab] OR "ehealth"[tiab] OR "m-health"[tiab] OR "e-health"[tiab] OR "text"[tiab] OR "text-based"[tiab] OR "internet"[tiab] OR "computer"[tiab] OR "computers"[tiab] OR "SMS"[tiab] OR "iphone"[tiab] OR "iphones"[tiab] OR "phone"[tiab] OR "phones"[tiab] OR "smartphone"[tiab] OR "smartphones"[tiab] OR "mobile"[tiab] OR "tablet"[tiab] OR "tablets"[tiab] OR "video game"[tiab] OR "video games"[tiab] OR "videogame"[tiab] OR "videogames"[tiab] OR "technology"[tiab] OR "technologies"[tiab] OR "web"[tiab] OR "online"[tiab] OR "social media"[tiab] OR "social network"[tiab] OR "social networks"[tiab] OR "email"[tiab] OR "e-mail"[tiab] OR "elearning"[tiab] OR "e-learning"[tiab] OR "telehealth"[tiab] OR "app"[tiab] OR "mobile app"[tiab] OR "mobile apps"[tiab] OR "applications"[tiab] OR "mobile application"[tiab] OR "mobile applications"[tiab] OR "mobile device"[tiab] OR "mobile devices"[tiab] OR "web based"[tiab] OR "web-based"[tiab] OR "cell phone"[tiab] OR "cell phones"[tiab] OR "cellphone"[tiab] OR "cellphones"[tiab] OR "smart phone"[tiab] OR "smart phones"[tiab]

#### Parents/caregivers

"family"[mesh] OR "parents"[mesh] OR "family health"[mesh] OR "caregivers"[mesh] OR "parent"[tiab] OR "parents"[tiab] OR "mother"[tiab] OR "mothers"[tiab] OR "father"[tiab] OR "fathers"[tiab] OR "guardian"[tiab] OR "guardians"[tiab] OR "caregiver"[tiab] OR "caregivers"[tiab] OR "parenting"[tiab] OR "parental"[tiab] OR "maternal"[tiab] OR "paternal"[tiab]

#### Race/ethnicity

"hispanic americans"[mesh] OR "african americans"[mesh] OR "ethnic groups"[mesh] OR "Emigrants and immigrants"[mesh] OR "Latinx"[tiab] OR "Latino"[tiab] OR "Latinos"[tiab] OR "Latina"[tiab] OR "Latinas"[tiab] OR "Hispanic"[tiab] OR "Hispanics"[tiab] OR "African American"[tiab] OR "African Americans"[tiab] OR "Black"[tiab] OR "Race"[tiab] OR "Racial"[tiab] OR "Ethnicity"[tiab] OR "Ethnicities"[tiab] OR "Minority"[tiab] OR "Minorities"[tiab] OR "minority health"[tiab]

#### Randomized controlled trial

(randomized controlled trial[pt] OR controlled clinical trial[pt] OR randomized[tiab] OR placebo[tiab] OR clinical trials as topic[mesh:noexp] OR randomly[tiab] OR trial[ti])

### Web of Science

#### Obesity

obes* OR BMI OR "body mass index" OR "obesity prevention" OR "body weight" OR overweight OR "weight status" OR "body fat" OR "prevent obesity" OR "healthy weight" OR adipos* OR "waist circumference" OR "body weight changes" OR anthropometry OR "over weight" diet OR "healthy eating" OR "energy density" OR "feeding behavior" OR "dietary habit" OR snack* OR "mealtime environment" OR meal* OR food OR "food habit" OR water OR "healthy snack" OR nutrition OR nutrient OR vegetable* OR fruit* OR juice OR "meal time behavior" OR "eating behavior" OR "food quality" OR "food preferences" OR "nutrition education" OR "nutrition quality" OR "dietary intake" OR consumption OR "whole grain" OR "sugar sweetened beverage" OR "eating habit" OR "improved access" OR availability OR intake OR "healthy eating habit" OR "healthy food" OR "improved consumption" OR "improved intake" OR "dietary quality" OR dietary OR "food and beverage consumption" "physical activity" OR active OR movement OR "active play" OR recess OR "play time" OR playtime OR "motor skill" OR sedentary OR exercise OR play OR "free play" OR "physical fitness" OR "motor activity" OR recreation OR "physical activities" OR "sedentary behavior"

#### Digital health

"digital health" OR mhealth OR ehealth OR "m-health" OR "e-health" OR text OR "text-based" OR internet OR computer OR computers OR SMS OR iphone OR iphones OR phone OR phones OR smartphone OR smartphones OR mobile OR tablet OR tablets OR "video game" OR "video games" OR videogame OR videogames OR technology OR technologies OR web OR online OR "social media" OR "social network" OR "social networks" OR email OR "e-mail" OR elearning OR "e-learning" OR telehealth OR app OR "mobile app" OR "mobile apps" OR applications OR "mobile application" OR "mobile applications" OR "mobile device" OR "mobile devices" OR "web based" OR "web-based" OR "cell phone" OR "cell phones" OR cellphone OR cellphones OR "smart phone" OR "smart phones"

#### Parents/caregivers

parent OR parents OR mother OR mothers OR father OR fathers OR guardian OR guardians OR caregiver OR caregivers OR parenting OR parental OR maternal OR paternal

#### Race/ethnicity

Latinx OR Latino OR Latinos OR Latina OR Latinas OR Hispanic OR Hispanics OR "African American" OR "African Americans" OR "Black" OR "Race" OR "Racial" OR "Ethnicity" OR "Ethnicities" OR "Minority" OR "Minorities" OR "minority health"

#### Randomized controlled trials

"clinical trial*" OR "research design" OR "comparative stud*" OR "evaluation stud*" OR "controlled trial*" OR "follow-up stud*" OR "prospective stud*" OR random* OR placebo* OR "single blind*" OR "double blind*"
